# Barbershops as a setting for supporting men's mental health during the COVID-19 pandemic: a qualitative study from the UK

**DOI:** 10.1192/bjo.2022.520

**Published:** 2022-06-27

**Authors:** Georgina Ogborn, Cerys Bowden-Howe, Patsy Burd, Maya Kleijn, Daniel Michelson

**Affiliations:** School of Psychology, University of Sussex, Brighton, UK

**Keywords:** COVID-19, community interventions, qualitative research, public mental health, gender

## Abstract

**Background:**

Previous research has highlighted the need to promote help-seeking by men with mental health problems.

**Aims:**

To investigate barbers’ views about offering mental health support for men in barbershops, with a specific focus on the psychosocial impacts of the COVID-19 pandemic.

**Method:**

We used a sequential mixed-methods qualitative design with online data collection. In Phase 1, 30 barbers in Southern England completed surveys exploring perceptions of their clients’ mental health during the COVID-19 pandemic, experiences of informal supportive roles and scope for providing formal mental health support in barbershops. Phase 2 involved member validation interviews and explored practice implications with three Phase 1 respondents.

**Results:**

Thematic analysis identified three overarching themes: ‘more than a haircut’ (describing how the physical and relational contexts of barbershops can offer a supportive environment for clients); ‘impacts of COVID-19’ (describing stressors related to the pandemic and implications for clients’ mental health and barber–client relationships); and ‘formal mental health strategies’ (describing opportunities for, and potential barriers to, formalising mental health support in barbershops).

**Conclusions:**

Barbers were aware of their clients’ worsening mental health during the COVID-19 pandemic. Barbershops were generally considered to be a suitable setting in which to promote good mental health, monitor for signs of mental ill health and provide information about local mental health services. Future work is needed to co-produce and evaluate formal mental health promotion and prevention strategies in barbershops. Particular attention should be given to service innovations that preserve the credibility and trust that are fundamental to the barbershop experience for many males.

Around one in eight men experience mental health problems^1^ and suicide is a leading cause of death among men globally.^2^ Unmet needs for mental healthcare among men have been reported extensively in prior research, with males under-represented in referrals to conventional talking therapies^3^ due to stigma, internalised role expectations and low mental health literacy.^4,5^ Evidence suggests that men may prefer supportive strategies that reframe help-seeking as reflecting conventionally masculine attributes such as ‘being brave’ and ‘in control’, allow for meaningful interpersonal connections, involve trusted providers and take place in familiar community settings.^5,6^

Public health initiatives linked to barbershops have shown promise in addressing disparities in health outcomes for men with conditions such as hypertension and prostate cancer^7,8^ and there is now growing interest in building partnerships between barbers and healthcare providers to deliver community-based mental health programmes. As well as being a frequently used community resource (e.g. males in the UK visit a barber every 2.5 weeks on average^9^), barbershops offer an environment in which clients can talk openly with their barbers about health and personal issues,^10,11^ connect with fellow clients^8^ and gain confidence in their appearance^12^. In many communities, interpersonal skills are seen as fundamental to the role, such that the customer should leave with ‘an uplifted spirit, happy, satisfied and feeling good about themselves’^13^ and conversations between barbers and clients can range from light-hearted fun to deeply meaningful and ‘quasi-therapeutic’.^10,11^

The evidence is less clear about the benefits of providing formal mental health support through barbershops. Various initiatives have been influenced by the ‘barbershop model’ of health promotion that emerged in the 1980s to address health problems in African-American communities, for whom barbershops have historically provided a safe gathering place with important social and cultural functions.^14^ Key components of the barbershop model have included training barbers to improve health literacy and offering guidance on how to initiate client referrals to professional healthcare providers. However, mental health outcome data have been scarcely reported and the applicability to other populations and contexts is uncertain, despite international interest and examples of public funding.^15^

The current study was concerned with understanding the barriers and facilitators to providing informal and formal mental health support for men in barbershops in the UK, with a particular focus on identifying promising approaches that could be applied during the COVID-19 pandemic and its aftermath. COVID-19 mortality rates have been significantly higher in males than females, and the prevalence of depression in males doubled during the first year of the pandemic.^16^ Disproportionate impacts have been borne by males from Black and minority ethnic groups, who have contracted and died from COVID-19 at significantly higher rates compared with White communities, as well as facing relatively higher levels of stress and mental health problems linked in part to precarious housing, employment and financial conditions.^17,18^

Expanding community-centred approaches to mental health provision is a cornerstone of the UK Government's ‘COVID-19 Mental Health and Wellbeing Recovery Action Plan’.^19^ Formative research is needed to explore how barbers can contribute to such efforts and to investigate how established informal support structures may be strengthened further, particularly given the disruptions to social networks caused by COVID-19 control measures. We investigated three research questions. First, how do barbers prefer to engage with their male clients in relation to mental health issues? Second, what mental health impacts have been observed by barbers during the COVID-19 pandemic? And third, what is the scope for providing formal mental health support in barbershops?

## Method

### Design

A sequential mixed-method qualitative design was used.^20^ A hybrid deductive–inductive approach was applied during a survey-based insight generation phase (Phase 1), followed by member validation interviews and exploration of practice implications (Phase 2). Mixed-method formative designs of this type have been used in other community-based mental health service research to enhance the richness of data available to inform community-based participatory interventions.^21^ All procedures involving human participants were approved prior to study commencement by the University of Sussex Ethics Committee (reference: ER/GO73/2). We assert that all procedures contributing to this work comply with the ethical standards of the relevant national and institutional committees on human experimentation and with the Helsinki Declaration of 1975, as revised in 2008. The study has been reported in line with consolidated criteria for reporting qualitative research (COREQ).^22^ A completed COREQ checklist is provided among the supplementary materials available at http://doi.org/10.1192/bjo.2022.520.

### Participants

**Fig. 1:**
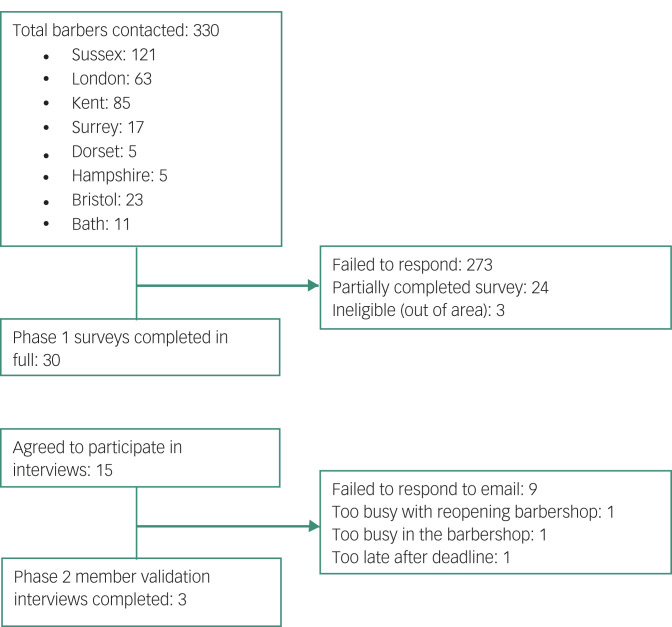
Participant flow diagram.

Eligible participants were male barbers working in barbershops catering exclusively or primarily to male clients in the South of England. In the first instance, we focused recruitment on neighbourhoods in the counties of East and West Sussex with relatively high levels of income deprivation.^23,24^ Starting in January 2021, Google Search was used to locate barbershops in relevant areas and these businesses were then contacted individually via telephone, email and Facebook accounts. Sampling was extended to other parts of Southern England to increase uptake in the latter stages of recruitment (March to April 2021). The survey completion target was 30, based on reasonable estimates of survey response rate in similar samples^25^ and taking into account the depth and detail of individual responses needed to address the research questions.^26^ We aimed to interview a smaller number of participants in Phase 2, where the priority was to engage in reciprocal discussions to help improve the credibility, validity and transferability of Phase 1 findings. Opportunity sampling was used to find barbers who were available to participate in interviews during April 2021. No prior relationship was established between the researchers and participants. The participant flow is summarised in Fig. 1.

### Measures

#### Phase 1

An 11-item online survey was created with ten open-ended free-text questions formulated in line with the study research questions (i.e. covering approaches to engaging with clients’ mental health, perceptions of clients’ mental health during the pandemic, and scope for providing formal mental health support to clients; see Supplementary File 1). An additional forced-choice question was used to assess barbers’ views about common daily stressors encountered by their clients during the pandemic, with response options adapted from an existing checklist (Brief Daily Stressors Screening Tool, BDSST^27^). The online survey also collected demographic information and included an ‘opt-in’ for respondents to be contacted about Phase 2.

#### Phase 2

Member validation interviews were planned in an online focus group format, but owing to scheduling difficulties were ultimately conducted as individual online interviews, using Zoom video-conferencing software. Open discussion was used to provide participants with opportunities to engage with, and add to, interpreted data from Phase 1, with specific prompts used to elicit implications for practice development (Supplementary File 2).

### Procedure

An outline setting out the study rationale and methods was sent to each identified barbershop's main email address or Facebook account. The message included weblinks to a detailed participant information sheet and consent form. Respondents who completed the consent form were automatically directed to the online survey. Those who additionally opted to take part in Phase 2 were contacted by email and offered a choice of times and dates for participating in an interview. Each interview was led by one female researcher while the other female researchers observed and took notes. The interviewers were all undergraduate psychology students at the time of data collection and had completed training in qualitative methods as part of their course; supervision was provided by a male clinical academic psychologist.

A summary of Phase 1 findings was presented at the beginning of each interview session. Following this, each participant commented and elaborated on interpreted data and practice implications. Interviews lasted for around 20 min, were audio-recorded and transcribed verbatim. No repeat interviews were required and transcripts were not returned to participants.

### Analysis

Thematic framework analysis followed a series of steps involving familiarisation, identifying a thematic framework, indexing, charting, and mapping and interpretation.^28^ This recursive process allowed the analysis to move back and forth through steps as required. Coding was guided by both predetermined categories (following logically from the research questions) and data driven codes.^29^ A preliminary coding frame was developed by the first four named authors and then applied independently to a subset of surveys, followed by comparison of codes and iterative revision of the frame using qualitative data from both phases until data saturation was achieved. Higher-order themes were refined further in light of Phase 2 interviews and in consultation with the senior author (D.M.). Results from the categorical survey question (related to commonly experienced stressors) were summarised using frequency counts and percentages and have been embedded within the wider thematic narrative below.

## Results

### Participant characteristics

Participants were drawn from across Southern England, with the majority (*n* = 22, 73%) from Sussex. Two-thirds of the participants cut men's hair exclusively and the mean age of their clients was distributed categorically as follows: 18–25 years (*n* = 11, 37%); 26–35 years (*n* = 15, 50%); and 36–45 years (*n* = 4, 13%).

### Thematic analysis

**Fig. 2:**
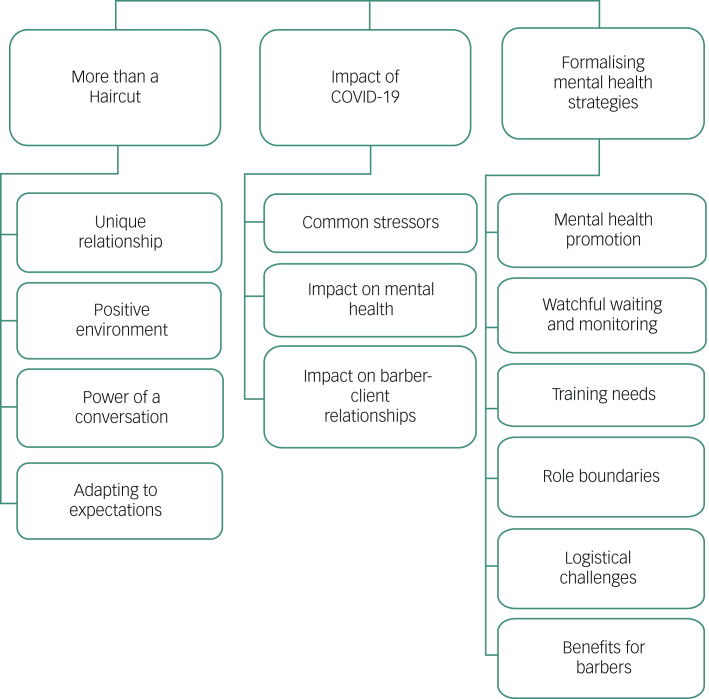
Matrix of study themes.

Figure 2 provides an overview of three over-arching themes and their sub-themes. These are elaborated in the narrative below and supported by illustrative quotes.

### More than a haircut

Barbers described how they provided a holistic service with a number of mental health-promoting benefits. The ‘unique relationship’ sub-theme emphasised the distinctive nature of the barber–client relationship, which was considered to be deeper than many other service provider–client relationships:

‘[Clients] become part of our barber community’ (Respondent 24, average (avg) client age 26–35)

‘You've a really impartial position on their lives’ (Respondent 10, avg client age 26–35).

The ‘positive space’ sub-theme reflected how the barbershop served as a welcoming and supportive space that offered an escape from daily life, and from which clients typically emerged with uplifted spirits and renewed confidence:

‘People come, they have a laugh, they have a joke, and they have a good time’ (Respondent 13, avg client age 18–25)

‘I always try and keep things upbeat; your client should leave feeling fresh not frantic after a visit’ (Respondent 10, avg client age 26–35)

‘A barbershop is a hub for men where they can relax, unwind, open up and distract themselves from the pressures of life’ (Respondent 23, avg client age 36–45)

‘The environment we create in the shop makes it a safe place for anyone to talk’ (Respondent 14, avg client age 26–35).

The ‘power of a conversation’ sub-theme captured how authentic and non-judgemental conversations are an especially valued aspect of the barbershop experience for many clients:

‘Speaking to someone with an unbiased opinion is important to them […] they open up about their worries or challenges and reach out’ (Respondent 23, avg client age 36–45)

‘Just listen and let someone be open, as talking is the best thing to do’ (Respondent 13, avg client age 18–25).

The ‘adapting to expectations’ sub-theme recognised how barbers adapted their interpersonal approach for different clients, some of whom may be less comfortable discussing personal matters:

‘Most clients just chat about their day, but for some it's an important opportunity to discuss something affecting their life’ (Respondent 22, avg client age 18–25)

‘A lot of guys just like to sit in silence for half an hour and relax’ (Respondent 18, avg client age 26–35)

‘There are different types of male customers […] some are happy to talk, some will completely blank you’ (Respondent 8, avg client age 18–25).

### Impacts of COVID-19

This theme encompassed descriptions of common stressors experienced by clients (sub-theme 1), mental health impacts for clients and barbers (sub-theme 2) and implications of the pandemic for barber–client relationships (sub-theme 3).

Direct health concerns related to COVID-19 (*n* = 9, 30%) were less commonly endorsed than social and economic impacts of the pandemic. In particular, ‘loneliness’ (*n* = 24, 80%) and ‘financial restrictions’ (*n* = 24, 80%) were the two most frequently noted sources of stress after ‘close persons’ (*n* = 25, 83%). Other reported categories of stressor were ‘dissatisfaction with occupation/education’ (*n* = 16, 53%), ‘other persons’ (*n* = 12, 40%) and ‘other health concerns’ (*n* = 7, 23%). Relatedly, barbers noted a strong pent-up demand for reconnecting with the social aspects of barbershops after COVID-19 public health restrictions affecting the industry were relaxed in mid-2020:

‘People after the first lockdown were more desperate to come back to have a conversation and see people’ (Respondent 11, avg client age 26–35)

‘We are here to make people feel good about themselves, haircut, chat, people have missed this over lockdown’ (Respondent 26, avg client age 26–35).

Barbers reported that many clients had revealed experiences of worsening mental health during post-lockdown barbershop visits:

‘Definitely more people are saying they're struggling with their mental health through COVID’ (Respondent 6, avg client age 36–45)

‘People are now putting their hands up and saying I've had it [worsening mental health], I've got it, I struggle with it and more people are coming out about it’ (Respondent 6, avg client age 36–45).

In some cases, clients’ mental health disclosures had aroused heightened concern from their barber and led to direct supportive efforts beyond the barbershop setting:

‘I had a client who was very much impacted by the lockdown that they thought about taking their own life […] I've kept contact […] and made them aware of the suicide prevention schemes’ **(**Respondent 1, avg client age 18–25).

Mental health impacts were also experienced by barbers themselves:

‘I struggled with mental health myself, I needed a purpose’ (Interviewee 3, London)

‘There are a lot of barbers in the industry that suffer from mental health [problems] […] it's a hard job to do when you're not feeling 100% yourself’ (Respondent 11, avg client age 26–35).

Notwithstanding renewed demand for barbers’ services, residual impacts of lockdowns extended to the atmosphere in some barbershops and the quality of barber–client relationships:

‘The barbershop isn't the same as it was a year ago, it's totally different now’ (Interviewee 2, Sussex)

‘Those regulars […] can't open up again or have lost that initial banter with you so it's almost like starting again really’ (Respondent 11, avg client age 26–35).

### Formalising mental health strategies

Many barbers recognised the potential to formalise mental health strategies in barbershops, although this was accompanied by concerns about overstepping role boundaries without appropriate training and in the context of competing workplace demands. The ‘mental health promotion’ sub-theme reflected suggestions about how barbers could play an important role in raising awareness of mental health problems and services, building on their existing position as informal providers of psychological support. Aside from passing on relevant information verbally during a haircut, suggestions were made about using posters and leaflets to share details of mental health services:

‘I think the NHS [National Health Service] could provide contact details that we can inconspicuously provide to a client that we may think needs help if they so wish’ (Respondent 14, avg client age 26–35)

‘It's about in that little time you are with someone cutting their hair, giving them the right places to go to get help’ (Respondent 12, avg client age 18–25)

‘It's about getting information out to them quick and giving them information on who is available for them to talk to straight away’ (Respondent 6, avg client age 36–45).

Some barbers had already taken concerted steps towards raising the profile of mental health in their settings:

‘Especially after COVID there is going to be a need for mental health support […] we can let them know we are here for them […] we need to get barbers involved quick’ (Respondent 6, avg client age 36–45)

‘We are going to be doing more with mental health in the shop, we are going to start putting stuff out there to show people we do care’ (Interviewee 2, Sussex).

The ‘watchful waiting and monitoring’ sub-theme related to suggestions that ongoing relationships with clients provide opportunities for monitoring mental health over time and for intervening sensitively at the right moment, particularly with clients for whom self-stigma may limit spontaneous help-seeking:

‘Show people we do care, we're not just there to cut their hair […] then people may open up a bit more’ (Interviewee 2, Sussex)

‘Just letting guys know it's ok to talk about things’ (Respondent 18, avg client age 26–35)

‘Double checking if he was okay and offering my ears if he needed to vent or talk about his mental health’ (Respondent 21, avg client age 26–35)

‘Be relaxed about it, don't go looking for it but being able to know what to say and how to help if you did come across someone who is crying out for support’ (Respondent 4, avg client age 26–35)

‘The largest stumbling block in regard to men's mental health is getting them to speak about it at all. Men tend not to discuss it’ (Respondent 17, avg client age 36–45)

‘They don't want to be seen as weak’ (Respondent 6, avg client age 36–45).

The ‘training needs’ sub-theme reflected barbers’ self-identified requirements for formal training in how to recognise mental health problems in clients and signpost them to appropriate services:

‘Giving barbers some training […] about the indicators that people are struggling with their mental health would be a good thing’ (Respondent 18, avg client age 36–45)

‘If we were trained, we would know how to approach the situation tactfully, to make sure you make things better and not worse’ (Interviewee 3, London)

‘If a barbershop wants to help with strategies to support mental health, I think a voluntary programme provided by the NHS in which professional clinicians can offer knowledge on warning signs/potential advice/do's and don'ts on what to say could help the industry’ (Respondent 14, avg client age 26–35).

Flexible training models were advocated in line with differing business models:

‘[Training should] definitely be different for every barbershop […] appointment-based barbershops would be better, compared to walk-in ones’ (Interviewee 1, Sussex)

‘What you think may work in one shop may not work in another’ (Respondent 13, avg client age 18–25).

A small number of barbers referred to existing training in suicide awareness (‘BarberTalk’):

‘Pushing more barbers to do this free course is definitely the way forward’ (Respondent 21, avg client age 25–35).

The ‘role boundaries’ sub-theme recognised concerns among some barbers about moving too far beyond their core professional role and making unwelcome incursions into clients’ private lives:

‘Clients don't need to be counselled by me’ (Respondent 12, avg client age 18–25)

‘There is only so much you can do as a barber unless you are trained […] but if you are trained you wouldn't be cutting hair as you would be a therapist’ (Interviewee 2, Sussex)

‘Not every barber is going to have the same view about it, some get it and want to help but some don't, and they avoid talking about mental health’ (Respondent 13, avg client age 18–25)

‘I don't feel I am responsible for helping people with their mental health’ (Respondent 2, avg client age 18–25)

‘I wouldn't want to break the trust of a client confiding certain details to me’ (Respondent 18, avg client age 26–35)

‘Some clients may hate the fact that you're suggesting they may need help. You don't want to lose customers’ (Respondent 9, avg client age 26–35).

The ‘logistical challenges’ sub-theme recognised that the demands of running a busy barbershop can limit scope for formalised mental health strategies:

‘Our appointment times are higher than the average barbershop, which means we have more time at our disposal to encourage meaningful conversations’ (Respondent 14, avg client age 26–35)

‘I haven't got the time to support people with their mental health’ (Respondent 2, avg client age 18–25)

‘Us barbers, we can't even think about doing anything else as it's so busy’ (Interviewee 2, Sussex)

‘They don't want to take that on as well as running a business, as running a business is stressful in itself’ (Interviewee 1, Sussex).

Physical space was also identified as a limiting factor:

‘We don't have a lot of space between clients and barbers so there would be a lack of confidentiality’ (Respondent 8, avg client age 18–25).

Finally, the ‘benefits to barbers’ sub-theme emphasised how supportive activities can have reciprocal benefits for those who work in barbershops:

‘If barbers were provided with workshops on topics related to mental health, we would not only be more well versed in how to handle potential situations effectively, it would lead to stronger client connections overall. So, from a business perspective, it's also a win’ (Respondent 10, avg client age 26–35)

‘From an employer's point of view, it's not just people coming into your barbershop, but also the people working in it too’ (Interviewee 1, Sussex).

## Discussion

This study investigated men's mental health during the COVID-19 pandemic from the perspective of barbers. Participants described how the physical and relational contexts of barbershops ordinarily contribute to a welcoming, non-judgemental and relaxed environment with a variety of mental health-promoting benefits. The loss of informal support available in barbershops was seen as being highly relevant during the pandemic, with study participants commonly reporting adverse mental health effects among their clientele and personally. Disruptions to normal social and economic functioning were recognised as major contributing factors to the suggested rise in mental ill health. Some barbers in the study had taken proactive steps towards formalising the support available through their workplaces, and others were open to such activities. However, concerns were also raised about overstepping professional boundaries and managing competing workplace demands.

Other studies have identified young adults, especially those from minority ethnic communities and those experiencing socioeconomic disadvantage, as a high-risk demographic for adverse mental health outcomes during the pandemic.^30^ Males in particular have been over-represented among emergency psychiatric presentations during periods of lockdown,^31^ whereas higher incidence of self-reported anxiety and depression has been found among females.^32^ There are also indications that men (at least from certain occupational groups) have been relatively less likely to seek formal help for mental health problems occurring during the pandemic.^33^

Barbers participating in the current study recognised that barbershops ordinarily provide men with a safe and familiar setting in which to socialise and discuss sensitive matters that may not be readily disclosed to others. Previous research has established that informal social interactions can enhance social belonging and cognitive functioning^34,35^ and that social connectedness is a strong and consistent predictor of positive mental health.^36^ There was consensus among participants that lockdowns, social distancing and other pandemic restrictions had made it harder to create supportive environments for clients. Future research could usefully explore whether and how barbers and their customers successfully reconnect, and the extent to which these relationships might have been permanently altered.

Many barbers in our sample were keen to explore formal strategies for supporting their clients’ mental health and they identified corresponding training needs in identifying and responding sensitively to signs of distress. Further suggestions were made about using printed materials and targeted verbal information to raise clients’ awareness of external services. We note that mental health promotion programmes designed for males in other community settings have often used gender-sensitive language that recognises men's interests and preferences. Examples include using the language of sport (e.g. ‘mental fitness’ and football metaphors) and emphasising ‘stress’ as opposed to ‘anxiety’ or ‘depression’.^6^ One study^37^ notably devised a ‘Man Card’ (sized like a conventional business card), which set out the steps for helping a friend and also listed external mental health resources.

A number of participating barbers in our study were concerned about overstepping role boundaries, potentially intruding into clients’ private lives and consequently losing business. Logistical challenges were also identified related to balancing mental health promotion and prevention activities with the daily requirements of running a business, particularly after periods of closure and fluctuating demand during the pandemic. Training activities should take these concerns into account to ensure feasibility and acceptability in the barbershop setting. Corresponding evaluations are required to demonstrate effects on barbers’ knowledge and behaviours, as well as downstream effects on clients’ mental health.

### Limitations

We acknowledge limitations relating to our methods of data collection and sampling. First, the study relied primarily on self-reported qualitative surveys, limiting the scope for elaborating on topics and clarifying participants’ responses. The use of survey methods was pragmatic given the prevailing restrictions on in-person meetings at the time of the study and scheduling limitations that restricted participants’ availability. These limitations also impacted on the participant validation phase (Phase 2), which was designed so that participants could engage with, and add to, survey data and its interpretation. In practice, Phase 2 coincided with a period when barbershops were due to reopen in England for the first time after a 10-week mandated closure and most of the Phase 1 survey participants were too busy to participate. Second, the validation interviews were intended to be conducted as focus groups, but scheduling constraints necessitated individual interviews. Third, the study was conducted solely from the perspective of barbers and we did not directly engage with their clients. Barbers in the study recognised that some customers were relatively open and comfortable with discussing their mental health, whereas others merely attended for a haircut. Future research should investigate the diversity of clients’ views to better understand what, how and for whom formal mental health support can be made available through barbershops. Specific attention should also be paid to opportunities and strategies for building social connections between users of barbershops and facilitating community action on a wider scale. This would be consistent with the important role played by social networks in strengthening individual and collective resilience more generally during the COVID-19 crisis.^38^

### Implications

This study was conducted against the backdrop of COVID-19 restrictions, at a time when psychosocial interventions led by community stakeholders have been an important focus of public policy and service development. Long recognised as a safe space for men to talk, barbershops provide an important locale for informal mental health support with the potential for building social connections and linking to formal public mental health systems. Given the emphasis placed on positive barber–client relationships in this context, it is vital that formal interventions should avoid potential pitfalls such as blurring professional boundaries and compromising confidentiality. Larger studies are now needed to elaborate how service innovations can effectively preserve the credibility and trust that are central to the barbershop experience for many males.

## Data Availability

The data used in this study are not publicly available owing to the conditions of participant consent.

## Supplementary material

For supplementary material accompanying this paper visit http://dx.doi.org/10.1192/bjo.2022.520. click here to view supplementary material
