# Supplementary material for: Barbershops as a setting for supporting men's mental health during the COVID-19 pandemic: a qualitative study from the UK
Source: BJPsych Open. 2022 Jun 27;8(4):e118. doi: 10.1192/bjo.2022.520 (PMC9237491; doi:10.1192/bjo.2022.520)
Supplement: Supplementary file 1 [file S2056472422005208sup001.zip › Supplementary_File_1.doc]

Supplementary File 1

**Template of Survey**

Barbershops as a setting for supporting men's mental health

**Barbershops as a setting for supporting men's mental health: a qualitative investigation in the COVID-19 context**

**INFORMATION SHEET**

**Invitation to take part**

You are being invited to take part in a research study to explore barbers' perspectives about how COVID-19 has affected their clients' mental health. Building on these insights, an optional second part of the study will look at the potential role of barbers in supporting community-based recovery from the secondary mental health impacts of COVID-19.

**Why have I been invited for testing and what will I do?**

We are interviewing barbers provided they have experience cutting male's hair in the South of England.  Our data gathering will take place in two stages.

1)    During the first stage you will answer an in-depth questionnaire to explore your impressions of clients and their mental health. We are interested in overall impressions and not personal information about your clients.

2)    If you also decide to take part the second stage, we will invite you to participate in an online group meeting using Zoom with up to 6 barbers. The meeting will summarise our findings from stage one and give you the opportunity to elaborate on previous points.

**What will happen to the results and my personal information?**

The results of this research may be written into a scientific report for a Psychology dissertation. We anticipate being able to provide a summary of our findings on request from the May 2021 (pb333@sussex.ac.uk, mk660@sussex.ac.uk, go73@sussex.ac.uk, cb618@sussex.ac.uk). Your anonymity will be ensured in the way described in the consent information below. Please read this information carefully and then, if you wish to take part, please sign to show you have fully understood this sheet, and that you consent to take part in the study as it is described here.    Thank you for carefully reading this information sheet, a copy of which you can keep for your records. This study is being conducted by a group of four undergraduate students from the School of Psychology, University of Sussex who are happy to be contacted if you have any questions.

CONSENT 
 
• I understand that by signing below I am agreeing to take part in the University of Sussex research described here, and that I have read and understood this information sheet. 
 
• I understand that my participation is entirely voluntary, that I can choose not to participate in part or all of the study, and that I can withdraw at any stage of testing without having to give a reason and without being penalised in any way (e.g., if I am a student, my decision whether or not to take part will not affect my grades). 
 
• I understand that an audio recording will be taken at the workshop stage of the study, for the use of examining data, which will then be destroyed at the end of the research. 
 
• I understand that since the study is anonymous, it will be impossible to withdraw my data once I have completed and submitted the test. 
 
• I consent to the processing of my personal information for the purposes of this research study. I understand that such information will be treated as strictly confidential (subject to legal limitations) and handled in accordance with the Data Protection Act 2018. 
 
• I understand that my collected data will be stored in a de-identified way (e.g. using ID numbers not names), and kept separate from other details about me (e.g. from the consent form). Electronic data will be stored on a password-protected computer, and hard-copies will be stored behind a locked door. 
 
• I understand that my identity will remain confidential in any written reports of this research, and that no information I disclose will lead to the identification in those reports of any individual either by the researchers or by any other party, without first obtaining my written permission. 
 
• I understand that I have given approval for the name of my workplace to be used in any written reports of this research. 

• I understand that personal details will be disclosed under codings to prevent my identity from being made public. 
 
• I understand that my name and data will not be shared with any third party outside the research group, unless I later provide written permission. 
 
• I understand that confidentiality cannot be guaranteed for information which I might disclose in the group interviews. 
 
Our research group at the University of Sussex carries out studies on mental health. Please click the consent box below if you are happy to partake in this study which will include the use of your data again in future studies if these have gained independent ethically approval, based on the strict confidentiality terms described above. 
 
Contact details: Patsy Burd, Maya Kleijn, Georgina Ogborn, Cerys Bowden-Howe pb333@sussex.ac.uk, mk660@sussex.ac.uk, go73@sussex.ac.uk, cb618@sussex.ac.uk

Q1 What is your sex?

- Male (1)
- Female (2)
- Non-binary / third gender (3)
- Prefer not to say (4)

Q2 What is the sex of your clients?

- Male (1)
- Female (2)
- Both (3)
- Other (5)

Q3 What is the average age of your clients?

- Under 18 (5)
- 18-25 (6)
- 26-35 (7)
- 36-45 (9)
- 46+ (10)

Q4 What is the postcode of your main workplace?

________________________________________________________________

**Please answer the following questions in as much depth as you can**

Q1. In what ways do you normally develop social connections with your clients?

________________________________________________________________

________________________________________________________________

________________________________________________________________

________________________________________________________________

________________________________________________________________

Q2. How important do you think these connections are to the mental health of your clients?

________________________________________________________________

________________________________________________________________

________________________________________________________________

________________________________________________________________

________________________________________________________________

Q3. When was the last time you became aware of a client's psychological needs? How did you respond and what difference do you think this made to your client?

________________________________________________________________

________________________________________________________________

________________________________________________________________

________________________________________________________________

________________________________________________________________

Q4. In what ways do your clients open up about their mental health to you and what changes, if any, have you noticed since the start of the pandemic?

________________________________________________________________

________________________________________________________________

________________________________________________________________

________________________________________________________________

________________________________________________________________

Q5. Are there any groups of clients that you think are particularly prone to mental health difficulties? How has this changed during the pandemic?

________________________________________________________________

________________________________________________________________

________________________________________________________________

________________________________________________________________

________________________________________________________________

Q6. Based on interactions with your clients since the start of the pandemic, which of the following stressors seem particularly important to their mental health? Click all that apply.

- Health concerns related to COVID-19
- Other health concerns (e.g. diseases, chronic sufferings)
- Close persons (e.g. family, household, friends, partner)
- Other persons (e.g. colleagues, fellow-students, neighbours, tenants, landlords)
- Dissatisfaction with education/occupation (e.g. examinations, work overload)
- Financial restrictions (e.g. low income, instalments)
- Loneliness
- Any others you have noticed? Please state below. ________________________________________________

Q7. How would you describe the role of barbershops in the local community? In what ways do you think this role has changed during the COVID-19 pandemic?

________________________________________________________________

________________________________________________________________

________________________________________________________________

________________________________________________________________

________________________________________________________________

Q8. What role(s), if any, do you think barbers can play in supporting the mental health of clients? Has your view of this changed as the pandemic evolves? If so, how?

________________________________________________________________

________________________________________________________________

________________________________________________________________

________________________________________________________________

________________________________________________________________

Q9. What is the potential for barbers to work together with NHS/other mental health professionals to identify and support vulnerable clients? What resources and approaches would be needed to make this work?

________________________________________________________________

________________________________________________________________

________________________________________________________________

________________________________________________________________

________________________________________________________________

Q10. Can you think of any challenges in developing mental health support through barbershops? What do you think is needed to overcome such challenges?

________________________________________________________________

________________________________________________________________

________________________________________________________________

________________________________________________________________

________________________________________________________________

Q11. What strategies could be used to engage barbers in developing mental health support through barbershops?

________________________________________________________________

________________________________________________________________

________________________________________________________________

________________________________________________________________

________________________________________________________________

Would you be interested in taking part in an online follow-up workshop discussing the trends we find to further our research at a later date?

- Yes - please provide your email address below so we can provide you with further details ________________________________________________
- No

**Barbershops as a setting for supporting young men's mental health: a qualitative investigation in the COVID-19 context**

Thank you for your responses.
If you have any complaints, concerns, or questions about this research, please feel free to contact us on 
pb333@sussex.ac.uk, mk660@sussex.ac.uk, go73@sussex.ac.uk, cb618@sussex.ac.uk

Any ethical concerns about the study may be directed to the ethics chair at crecscitec@sussex.ac.uk 

However, once the questionnaire is submitted, the right to withdrawal is no longer possible. We have gathered your contact details in a way that does not link them to your responses. 

 Finally, thank you again for helping us with this research.
