# Supplementary material for: Barbershops as a setting for supporting men's mental health during the COVID-19 pandemic: a qualitative study from the UK
Source: BJPsych Open. 2022 Jun 27;8(4):e118. doi: 10.1192/bjo.2022.520 (PMC9237491; doi:10.1192/bjo.2022.520)
Supplement: Supplementary file 1 [file S2056472422005208sup001.zip › Supplementary_File_2.doc]

Supplementary File 2

**Member Validation Schedule**

All interviews were led by one main researcher throughout

**Welcome**

**167157 to say**: Hi thank you so much for coming today.

Firstly we would like to thank you so much for completing the first part of the study and now attending this validation interview. Your time is really highly appreciated.

My name is (167157), and I would like to introduce you to my dissertation partners:

***researcher** **unmutes**: Hi my name is (researcher) thank you very much for joining, we are just here to observe and take notes.

***researcher** **unmutes**: Hi, my name is (researcher) nice to see you all.

***researcher** **unmutes**: Hi my name is (researcher).

**167157 to say**: Just as a reminder, we are a group of Psychology students studying at Sussex University working on our dissertation. Our research is based around barbershops as a setting for supporting males mental health and we’re exploring the views of barbers around the time of the COVID-19 pandemic. We have invited you to join today to get some clarifications on our findings from the first stage of the study.

**Audio**

**167157 to say:** Please can we have your permission to audio record this interview in case we miss some important feedback or information?

**Step 1: Summary of findings:**

**167157 to say:** As a group we have thematically analysed the data from the 30 barbers who took part in the first stage of the study and we have interpreted the results by grouping them into individual themes:

- More than a haircut
  - Relationship
  - Gauging on client’s mood
  - Free of judgement (unbiased opinion)
- Barbershop setting
  - Safe environment for men
  - Sit and talk
- Every individual client and barber different
  - Some open some not
  - Some barbers did not know much about mental health, some not willing to support, some barbers suffering themselves
- Pandemic impacted mental health
  - Stressors
- Men’s mental health (help-seeking behaviour)
  - Stigma
  - Struggle to open up
- Intervention strategies
  - Training barbers
  - Linking with other services
  - Raise awareness
- Practical barriers
  - No time (busy)
  - Confidentiality
  - Wearing a mask
  - Can’t greet them the same way

**Step 2: Discussion**

**167157 to say:** “Based on these findings, does this feel authentic to your experience and please feel free to add any information you believe we may have missed?”

**Step 3: Clarification of main findings**

**167157 to say:** “We found that a lot of the barbers we spoke to suggested ‘training’ would be a good strategy to engage barbers in developing mental health support through barbershops :”

- How would this look like in practise?
- What should ‘training’ try to do?
- What should ‘training’ not do?
- Who should give the ‘training’?
- How do we interest barbers in ‘training’?
- Would certain types of barbers need different training or would it be the same for everyone?
- What are other potential barriers around these ideas?
- What are the potential solutions to these barriers that can be taken forward for the future?

**Final question**

**167157 to say:** What is one thing you have learned from this experience, that you will take back to work with you?

**167157 to say**: Thank you for coming
